# Does improved access to diagnostic imaging results reduce hospital length of stay? A retrospective study

**DOI:** 10.1186/1472-6963-10-262

**Published:** 2010-09-06

**Authors:** Petter Hurlen, Truls Østbye, Arne S Borthne, Pål Gulbrandsen

**Affiliations:** 1 Helse Sør-Øst Health Services Research Centre Akershus University Hospital Sykehusveien 27, NO-1478 Lørenskog, Norway; 2 Centre for Diagnostic Imaging Akershus University Hospital Sykehusveien 27, NO-1478 Lørenskog, Norway; 3 Department of Community and Family Medicine Duke University Medical Center 318 Hanes House, DUMC 2914 2914 Durham, NC 27710 USA; 4 Duke-NUS Graduate Medical School Singapore 2 Jalan Bukit Merah 169547, Singapore; 5 Faculty Division Akershus University Hospital University of Oslo Forskningsveien 3 A, NO-0316 Oslo, Norway

## Abstract

**Background:**

One year after the introduction of Information and Communication Technology (ICT) to support diagnostic imaging at our hospital, clinicians had faster and better access to radiology reports and images; direct access to Computed Tomography (CT) reports in the Electronic Medical Record (EMR) was particularly popular. The objective of this study was to determine whether improvements in radiology reporting and clinical access to diagnostic imaging information one year after the ICT introduction were associated with a reduction in the length of patients' hospital stays (LOS).

**Methods:**

Data describing hospital stays and diagnostic imaging were collected retrospectively from the EMR during periods of equal duration before and one year after the introduction of ICT. The post-ICT period was chosen because of the documented improvement in clinical access to radiology results during that period. The data set was randomly split into an exploratory part used to establish the hypotheses, and a confirmatory part. The data was used to compare the pre-ICT and post-ICT status, but also to compare differences between groups.

**Results:**

There was no general reduction in LOS one year after ICT introduction. However, there was a 25% reduction for one group - patients with CT scans. This group was heterogeneous, covering 445 different primary discharge diagnoses. Analyses of subgroups were performed to reduce the impact of this divergence.

**Conclusion:**

Our results did not indicate that improved access to radiology results reduced the patients' LOS. There was, however, a significant reduction in LOS for patients undergoing CT scans. Given the clinicians' interest in CT reports and the results of the subgroup analyses, it is likely that improved access to CT reports contributed to this reduction.

## Background

The implementation of a Radiology Information System (RIS) and a Picture Archiving and Communication System (PACS), and the integration of these systems with the Electronic Medical Record (EMR), may improve the use of diagnostic imaging in clinical practice. This Information and Communication Technology (ICT) can reduce the radiologists' reporting time, and make the reports and images instantly available to clinicians hospital-wide [1-10].

In May 2005, RIS and PACS (Siemens MagicSAS ^® ^and MagicView ^®^, Erlangen, Germany) were introduced to radiologists at a Norwegian five-hundred bed university-affiliated hospital. Both systems were integrated with the EMR (DIPS EPJ ^®^, Bodø, Norway). This complete technology shift will be referred to below as 'the ICT introduction'. Before the ICT introduction, radiologists read images on film. Clinicians had to walk to the Radiology Department to look at these images. Reports were printed and distributed on paper. For emergency ultrasound (US) cases, handwritten summaries accompanied the patients returning to the wards.

After the ICT introduction, images were immediately (within five minutes) available hospital-wide to clinicians with legal access to the patient's record. All radiology reports were entered directly into the EMR as soon as they were finished (also within five minutes). The reports were issued in two versions: a preliminary version after one radiologist's examination of the images, and a final version once a specialist in radiology had verified the conclusion.

In a previous study of the impact of this ICT introduction, we observed that the radiology turnaround time (RTAT), i.e. the time from the examinations until the reports were completed, was reduced after one year [11]. For preliminary reports, the median RTAT was reduced from 13.4 to 2.7 hours. For final reports, median RTAT was reduced from 22.6 to 15.1 hours. Two years after the ICT introduction, the RTAT for final reports was back to the pre-ICT level, and has, for various reasons, continued to increase. The RTAT for preliminary reports also increased somewhat, except for preliminary CT reports.

In a study of clinicians' use of the reports in the EMR, we observed that clinicians read reports soon after they were available [12]. The median time from a preliminary report becoming available in the EMR until it was opened was 0.8 hours for Computed Tomography (CT) reports, and 1.1 hours for Computed Radiography (CR) reports. Significantly more of the CT reports than CR reports were read (55% vs. 36%, p < 0.01). For final reports, the median time was 3.3 hours for CT and 3.5 hours for CR. Significantly more final CT reports were read than CR reports (91% vs. 87%, p < 0.01). Before the ICT introduction, the median time until the result was presented during a radiology round - a meeting between clinicians and radiologists - was 18 hours. However, important results were often communicated orally.

The Magnetic Resonance Imaging (MRI) service was limited and varied somewhat during the observation periods. MRI reporting was consequently not studied separately. The Department did not offer MRI examinations of emergency cases.

The capacity for performing the diagnostic imaging examinations did not change between the two periods.

The objective of the current study was to assess whether the improvements in radiology reporting and clinical access to diagnostic imaging information one year after the ICT introduction were associated with a corresponding reduction in the length of patients' hospital stay (LOS).

## Methods

Approval for this study was obtained from the Norwegian Social Science Data Service (NSD) and the Regional Ethics Committee, and the Duke University Medical Centre Institutional Review Board exempted this study from review.

Data relating to all hospital stays for all patients discharged between February 1^st ^and 28^th ^2005 and between February 1^st ^and 28^th ^2006 were retrieved from the hospital EMR. These periods were chosen because of the documented improvement in availability and access to the results of diagnostic imaging.

Patients from psychiatric and geriatric wards were excluded. All other patients were included, even if they had been admitted and discharged the same day. The data set included the date and time of admission and discharge, discharge diagnoses, number and categories of imaging examinations, and the clinical department responsible for the patient. LOS was calculated from the admission and discharge time stamps.

We did not have a strong a priori hypothesis as to how the different aspects of the ICT introduction would influence clinical practice, and thereby length of stay, or if there would be differences between different modalities, patient groups or clinical departments. Rather than creating hypotheses through deduction or by performing a feasibility study, our hypothesis generation was assisted by a data splitting approach. This approach guards against (unintended) hypothesis fishing, and ensures the integrity of the computed p-values [13]. The data set was randomly split into two parts. The first part, the exploratory data set (33%), was used to assist in generating the hypotheses. The remaining data, the confirmatory data set (67%), was used to test the hypotheses. The reported p-values for changes in LOS for each modality and for the whole patient group are based on the confirmatory data set. Once a hypothesis had been verified, the complete data set was used for quantification, and is presented in the figure and tables. The purpose of splitting data in this way was to ensure that our statistical tests were performed on data that were not used to generate the hypotheses.

Changes in LOS within each subgroup (e.g., Tables 1 and 2) were analyzed using the two-sided non-parametric Mann-Whitney U-test. To compare changes in LOS between subgroups (Table 2), an independent sample t-test was used. The change in the number of examinations was analysed using the chi-square test. The significance levels (predetermined at α < 0.05) are reported. SPSS (v. 15.0, ^© ^SPSS Inc.) was used for data management and analysis.

**Table 1:** LOS before and after the ICT introduction for all patients with one or more imaging diagnostic examinations.

	Stays	Mean	Median	SE
Pre-ICT	4,244	3.50 days	1.72 days	0.08 days
Post-ICT	4,648	3.34 days	1.50 days	0.08 days
				*p = 0.43*

**Table 2:** LOS before and after ICT for patients with discharge diagnoses recorded in both periods in both groups.

	**Non CT**				**CT**			
	**Stays**	**Mean**	**Median**	**SE**	**Stays**	**Mean**	**Median**	**SE**
	
Pre-ICT	1,509	1.96 d	0.56 d	0.10 d	194	8.42 d	5.46 d	0.68 d
Post-ICT	1,579	1.84 d	0.56 d	0.09 d	255	6.06 d	3.00 d	0.54 d

All durations are in days. The reduction in LOS for the CT group was significant alone (p < 0.03) and when compared to the non-CT group (p < 0.01).

## Results

The study included 8,892 hospital stays. A total of 1,275 different primary discharge diagnoses were used (International Classification of Diseases, ICD-10). Most patients, 57.6%, were discharged without receiving any diagnostic imaging examinations. CR was the most frequent diagnostic imaging examination: during their hospital stay, 35.2% of patients had one or more CR examination, whereas 11.8% of the patients had one or more CT scans. The Neurology department was the most frequent user of CT - 36.1% of their patients had one or more scans, while 18.7% of general surgery patients and 14.7% of orthopaedic surgery patients had CT scans. US examinations were performed for 7.4% of the patients, while only 2.5% received MRI scans.

The mean and median LOS before and after the ICT introduction are presented in Table 1. There was no significant reduction in LOS (p = 0.43 in the confirmatory data set). However, the exploratory analysis indicated a significant reduction in LOS for one subgroup of patients - patients who had one or more CT scans during their hospital stay. This was verified by the confirmatory data set.

**Figure 1:**
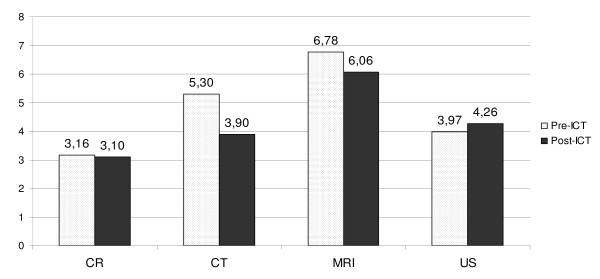
**Median LOS before and after ICT by modality (in days)**.

Figure 1 shows the median LOS for the main modality groups. The median in-patient stay for patients with CT scans was significantly shorter (p < 0.04) after the ICT introduction (3.9 days) than before (5.3 days), a 26% reduction. The reduction in LOS for the CT patient group was also significant compared to patients who received no CT scans during their hospital stays (p < 0.05). There was a reduction in LOS for MRI and CR patients, and an increase in LOS for US patients. These changes were, however, not statistically significant.

The clinical departments and support units were asked to identify major improvements in procedures or routines between the two observation periods. None were reported that should have had a significant impact on LOS. The data set was also investigated by referring unit, but there were no significant changes for any individual clinical department. There was no statistically significant increase in the number of patients with CT scans from the pre-ICT to the post-ICT period.

The CT patient group was heterogeneous, as 445 different primary discharge diagnoses were used for these 1,049 patients. To reduce the impact of this heterogeneity and to reduce the impact of any diagnostic routine changes not reported by the clinicians, a subset of patients were selected based on discharge diagnosis. We only included diagnostic patient groups where some - but not all - had CT scans in the pre-ICT period, and some - but not all - had CT scans in the post-ICT period. This would exclude diagnostic patient groups that was examined by CT only in one of the periods, and the effect of any diagnostic improvement would apply to both CT and non-CT patients alike. This subgroup consisted of 3,537 patients with 59 different discharged diagnoses (presented in Table 2). The changes in LOS was first analysed separately for CT patients in this subgroup, and then compared to the non-CT patients. There was a significant reduction (p < 0.03) in LOS for CT patients in this subgroup. The reduction was also significant (p < 0.01) when comparing the CT to the non-CT patients.

## Discussion

In previous studies of the ICT introduction [11,12] we found a RTAT reduction from 13.4 to 2.7 hours for preliminary, and from 22.6 to 15.1 hours for final reports. We also observed faster and more comprehensive clinical access to the results of diagnostic imaging after the introduction of RIS and PACS, and the integration of these with the EMR system. The current study did not reveal a corresponding significant reduction in LOS (Table 1). This finding is similar to those reported by others [2,14,15].

However, we found a significant reduction in LOS for one group of patients - patients who had undergone CT scans (Figure 1). Our previous studies indicated a particular clinical interest in the radiologists' CT scan reports. CT scans have become an important part of modern medicine, and provide detailed information that cannot be acquired more efficiently in other ways. It is therefore possible that some clinical decisions are delayed until the results of these scans are available, and that earlier availability of results can lead to earlier clinical actions which, in turn, lead to improved patient care and earlier discharge. CT is often also used to exclude serious conditions, and patients frequently wait for a negative CT examination report before being discharged. In this study we observed a significant reduction in LOS for CT patients both between the pre-ICT and post-ICT periods, and relative to patients who did not undergo CT examinations. It is likely that the ICT-enabled improved access to CT reports is responsible for at least part of this reduction.

The reduction in LOS for the MRI patients was not statistically significant. Only 2.5% of the patients had an MRI scan, and it is possible that this sample size was too small to demonstrate an actual reduction. However, it is also possible that the ICT introduction did not reduce LOS for this patient group. As MRI capacity was limited, MRI was primarily used for complex cases where the result of various clinical examinations would be compared before any diagnostic conclusion was made. Also, in non-emergency cases, patients would frequently be discharged before the results of the various examinations were available, with a scheduled follow-up in the outpatient department to make the final diagnostic conclusion. In such cases, improved access to reports would not influence LOS.

CR was the most frequent type of diagnostic imaging in this study. In our experience, CR is mostly used to supplement clinical examinations and laboratory tests, and decisions will rarely be postponed by a delayed radiology report. For example, a patient with clinical signs of pneumonia may get antibiotics before the results of the radiographs are available. Orthopaedic surgeons often prefer to interpret the images directly, rather than wait for the radiologist's opinion. In addition, many CR results are negative. We would therefore not expect a major reduction in LOS for patients examined with this modality even with improved access to the radiologist's diagnostic reports. The observed small reduction was not statistically significant.

For US patients, the observed increase in LOS was not significant. In the pre-ICT period, handwritten reports with the main conclusions from the examination accompanied emergency patients to the ward or clinical examination room. The clinicians would consequently have the radiologists' opinion available at least as early as with the post-ICT routines, and they could read the results without having to log on to the EMR. This may outweigh any effect the ICT introduction may have had on routine cases.

Nitrosi et al. [8] reported a reduction in LOS after PACS implementation. The reduction was largest for neurology patients. In our study, the neurology department was the most frequent user of CT scans. However, whether we included all neurology patients or only the subset of neurology patients that had undergone any form of imaging diagnostics, the reduction in LOS we observed was not significant. The neurology patients constituted, however, only 7.5% of the total patient group.

Watkins et al. [14] studied the influence of PACS on the LOS for two specific surgical procedures (total hip replacement and total knee replacement). They observed a 25% reduction for one of the procedures and no reduction for the other. They concluded that it was likely not a true PACS effect.

There were no other major changes in the hospital organisation or diagnostic approach between the two observation periods, neither according to clinicians' reports nor from our experience. However, new procedures, new routines and new approaches are continuously introduced, and clinicians may have forgotten about changes that could have influenced the LOS. In our study we have tried to compensate for the ever-changing environment in two ways. First, we included all somatic patients. For CT patients, 445 different diagnoses were used as discharge diagnoses, and even though new routines may have been introduced for some of these diagnoses despite the reports from the clinicians, most clinical procedures were probably unchanged after one year. Second, we selected a subset of patients with discharge diagnoses used for patients in all four categories; for patients before and after the ICT introduction, both with and without CT scans (Table 2). This should reduce the impact of changes in routines relating to specific diagnoses, as they would apply to both the CT and non-CT groups. This would also reduce the effect of the diagnostic heterogeneity. As indicated, the reduction in LOS was significant also for CT patients in this reduced patient group. Because of the large variety in diagnoses, we have not analysed specific diagnostic groups or adjusted for diagnostic variations.

It should be noted that we compared heterogeneous groups. The mean LOS for patients without CT scans was less than three days, and six to seven days for CT patients. For the reduced set of diagnoses, the mean for patients without CT scans was less than two days, and six to eight days for CT patients.

There is a strong financial pressure to increase productivity and reduce LOS - the average LOS for all hospitals in Norway was reduced from 5.1 days in 2005 to 5.0 in 2006 [16]. It is likely that such pressure has a greater impact on patients with longer hospital stays. Most of the patients who received CT scans were in this group.

## Conclusion

Our study showed that even with an ICT-enabled improved clinical access to the results of diagnostic imaging, we could not identify a corresponding reduction in the length of hospital stay when all patients were considered together. However, one subgroup of patients, namely those with CT scans, had 25% shorter hospital stays after the introduction of RIS and PACS, and the integration of these systems with the EMR. Given the clinicians' particular interest in CT reports it is likely that this reduction in length of hospital stay in part was caused by the improved clinical access to these reports. New clinical routines and a general drive towards efficiency may also have contributed to the result.

## List of abbreviations

CR: Computed Radiography; CT: Computed Tomography; EMR: Electronic Medical Record; ICT: Information and Communication Technology; LOS: Length Of patient's hospital Stay; MRI: Magnetic Resonance Imaging; PACS: Picture Archiving and Communication System; RIS: Radiology Information System; RTAT: Radiology report Turnaround Time; US: Ultrasound

## Competing interests

The authors declare that they have no competing interests.

## Authors' contributions

PH originated the idea for this study and prepared the manuscript. PH and PG performed the statistical analysis. All authors participated in the design of the study and interpreted the data, and all have read and approved the final manuscript.

## Pre-publication history

The pre-publication history for this paper can be accessed here:

http://www.biomedcentral.com/1472-6963/10/262/prepub
